# Visceral Adiposity and Lower-Body Strength and Endurance in Women: Correlations Using BIA and the Chair Stand Test

**DOI:** 10.3390/healthcare13212767

**Published:** 2025-10-31

**Authors:** Nouf Abdulaziz Aljawini

**Affiliations:** Department of Community Health Sciences, College of Applied Medical Sciences, King Saud University, Riyadh 11433, Saudi Arabia; naljawini@ksu.edu.sa

**Keywords:** visceral adipose tissue (VAT), sarcopenic obesity, muscle function, lower body strength, endurance, BIA, 30CST, menopause

## Abstract

**Background:** Visceral adipose tissue (VAT) around internal organs is strongly related to metabolic disorders. While its metabolic effects are well-established, its influence on musculoskeletal function, particularly lower-body strength and endurance in women, remains underexplored. Lower-body strength is essential for mobility, independence, and fall prevention. The 30 s chair stand test (30CST) is a reliable measure of lower-body function, and bioelectrical impedance analysis (BIA) offers a non-invasive method for evaluating VAT. Despite its potential, BIA remains underutilized in clinical practice. Integrating these tools could provide critical insights into how VAT affects functional health and guide evidence-based interventions. **Objective:** To examine the relationship between visceral adiposity, quantified by visceral fat rating (VFR) via BIA, and lower-body strength and endurance assessed by the 30CST in women. **Methods:** A cross-sectional study of 131 Saudi women examined VAT using BIA with VFR as a VAT marker. Lower-body strength and endurance were evaluated using the 30CST. Spearman’s rank correlation was employed to explore relationships between VFR and 30CST. **Results:** The median age was 56 (IQR 45–61). The median VFR was 10 (IQR 7–12), and the median 30CST score was 8 (IQR 7–10). In the entire sample, a significant negative correlation was observed between VFR and 30CST performance (r = −0.4106, *p* < 0.0001). Women with obesity (n = 73) had significantly higher VFR (12, IQR 10–13) compared to women without obesity (n = 58), who had a median VFR of 7 (IQR 6–9) (*p* < 0.0001). In contrast, women with obesity had significantly lower 30CST (8, IQR 6–9) compared to those without obesity (9, IQR 8–11) (*p* = 0.0004). Additionally, the entire sample had significant negative correlations between 30CST and age, weight, BMI, %BF, FM, and FFM (*p* < 0.05). **Conclusions:** Elevated visceral fat is associated with lower lower-body strength and endurance in women, highlighting the value of routine visceral fat assessment for guiding musculoskeletal health evaluation and management.

## 1. Introduction

Visceral adipose tissue (VAT) refers to the adipose tissue located within the abdominal and thoracic cavities, surrounding internal organs. It is anatomically divided into thoracic depots (including pericardial, epicardial, non-pericardial, and perivascular fat) and abdominal depots (comprising intraperitoneal and retroperitoneal fat) [1].VAT plays a critical role in the pathophysiology of several metabolic disorders, including cardiovascular diseases, type 2 diabetes mellitus, and insulin resistance, largely due to its secretion of pro-inflammatory adipokines and the dysregulation of lipid and glucose metabolism [1,2].

In women, particularly after menopause, VAT is a significant determinant of metabolic dysfunction [3,4]. VAT accumulation follows a distinct pattern in women, with levels increasing until approximately the age of 60, and then declining in those over 70 [4]. Particularly, women more than a decade past menopause tend to exhibit elevated VAT levels and a higher VAT-to-subcutaneous adipose tissue (SAT) ratio [4]. Furthermore, ethnic variations in VAT distribution are evident, with Hispanic women demonstrating the highest levels of visceral fat accumulation [4].Given the substantial impact of VAT on metabolic and cardiovascular health [2], it is imperative to assess VAT independently of traditional anthropometric measures, such as body mass index (BMI) and waist circumference, which do not accurately reflect visceral fat distribution [2,4].

Despite extensive research investigating the association between VAT and cardiometabolic risk factors, the effect of visceral adiposity on musculoskeletal function remains underexplored [2,3]. Adipokines and myokines mediate bidirectional crosstalk between muscle and adipose tissue, playing essential roles in regulating both metabolic homeostasis and functional capacity [5]. While much of the clinical research has focused on the effects of exercise on VAT reduction or the metabolic consequences of sarcopenia in obesity, there has been insufficient attention to the impact of VAT on lower-body muscle function, particularly strength and endurance, which are essential for daily activities [5,6,7,8].

Additionally, the literature on sarcopenic obesity has predominantly focused on total body fat rather than VAT, often using handgrip strength (HGS) as a surrogate measure of muscle strength [6,9,10,11].

This approach overlooks the critical contribution of lower-body muscle function to mobility, independence, and overall health [9].

Bioelectrical impedance analysis (BIA) is a widely utilized, non-invasive method for assessing body composition, including fat mass, lean mass, and total body water. Despite its extensive clinical use for these parameters, BIA’s potential to evaluate visceral adipose tissue (VAT)—a key determinant in the pathophysiology of metabolic and cardiovascular diseases—remains underexploited [12]. Advanced imaging modalities such as magnetic resonance imaging (MRI), computed tomography (CT), and dual-energy X-ray absorptiometry (DEXA) provide precise quantification of VAT. Still, high costs, limited availability, and the need for specialized interpretation constrain these techniques [1,2,3]. In contrast, BIA offers a more accessible, cost-effective alternative for assessing VAT, making it particularly valuable in clinical settings [12].

Previous research has highlighted the high prevalence of obesity and reduced muscle strength in Saudi women [6,7,8], with handgrip strength (HGS) values significantly lower than international reference standards [6]. This indicates the importance of further examining the role of visceral adiposity in muscle function. Lower-body strength and endurance, critical components of functional capacity and fall risk [13], can be effectively measured using the 30 s chair stand test (30CST), which is strongly correlated with mobility and independence [14,15,16,17,18].

This study’s hypothesis is that increased visceral fat, as measured by BIA, negatively correlates with lower-body strength and endurance, as assessed by the 30CST, in women. The main research question is: How does visceral adiposity, quantified through BIA, influence lower-body strength and endurance, as evaluated by the 30CST, in women? This investigation aims to understand better the relationship between VAT and musculoskeletal function, emphasizing the clinical importance of VAT assessment in optimizing health outcomes and preventing falls in women.

## 2. Methods

### 2.1. Design and Settings

This cross-sectional study followed the STROBE guidelines [19] and was conducted at Riyadh’s King Saud University Medical City (KSUMC) clinics from August 2023 to March 2025. The Institutional Review Board of the KSU College of Medicine (No. E-21-5998) obtained ethical approval, and all participants provided written informed consent. The study adhered to the ethical principles outlined in the Helsinki Declaration for research involving human subjects [20].

### 2.2. Participants and Protocol

Women who visited the outpatient clinics at King Saud University Medical City (KSUMC) and completed the study procedures were eligible to participate. Participants were recruited using a non-probabilistic convenience sampling method. A total of 131 women aged 18 to 94 were enrolled in the study. Participants’ medical histories were verified using their medical records. Inclusion criteria included Saudi women aged 18–94 who were ambulatory and able to complete the study assessments. Exclusion criteria included pregnancy, acute medical conditions or treatments that could affect body composition or strength, and physical disabilities that might interfere with bioelectrical impedance measurements or strength assessments.

For this investigation, obesity was defined as a body mass index (BMI) of 30 kg/m^2^ or higher [21]. The Chair Stand Test is a validated assessment of lower-body strength, conducted per EWGSOP2 sarcopenia screening guidelines [22]. All participants completed it safely and independently. All study procedures were conducted by a certified clinical researcher, following established standard operating procedures (SOPs).

### 2.3. Clinical Procedures

#### 2.3.1. Assessment of Anthropometrics, Body Composition, and Bioelectrical Impedance Analysis (BIA) Protocols

A trained researcher conducted anthropometric measurements and body composition assessments adhering to the standard operating procedures (SOPs) outlined in the study by Aljawini and Habib (2023) [6]. The estimation of body composition was performed using bioelectrical impedance analysis (BIA) with a Tanita MC-980 device (Tanita, Tokyo, Japan). This analyzer uses multi-frequency bioelectrical impedance to estimate body composition across five segments: both arms, both legs, and the trunk. It operates at six frequencies (1 kHz to 1 MHz) to differentiate tissue components such as fat, muscle, protein, minerals, and total body water. The device employs an 8-electrode system, delivering electrical current through the fingertips and toes while measuring voltage at the palms and heels. During assessment, participants stood barefoot on footplate electrodes and held hand grips with integrated sensors. Segmental analysis was used to evaluate lean mass distribution across each region. Body fat percentage (%BF) and fat-free mass (FFM) were derived from these measurements. All assessments were performed by a trained researcher following standardized procedures reported in established protocol [6].

#### 2.3.2. Assessment of Visceral Fat Using the Visceral Fat Rating (VFR) Index via Bioelectrical Impedance Analysis (BIA)

The Visceral Fat Rating (VFR) is an index quantifying visceral fat. It is estimated indirectly through Bioelectrical Impedance Analysis (BIA), with results expressed as a score ranging from 0 to 59 (without units). Scores between 1 and 12 indicate a healthy amount of visceral fat, while scores from 13 to 59 indicate excessive visceral fat [23,24]. This BIA technology has recently been validated for adults with varying physical activity levels [25].

#### 2.3.3. Assessment of Lower Body Strength and Endurance via the 30-Second Sit-to-Stand Test (30CST)

The 30-Second Sit-to-Stand Test (30CST), also known as the 30-Second Chair Stand Test, was employed to assess lower-limb strength and endurance in the women participating in this study. As part of the Fullerton Functional Fitness Test (FFT) battery, the test measured the number of sit-to-stand repetitions each participant could complete within 30 s [15,26].This test provided a reliable and objective measure of lower-body functional capacity, essential for performing everyday activities, such as rising from a chair or standing up from a seated position [15,18].

The 30CST was specifically chosen due to its strong correlation with fall risk, making it a crucial tool for evaluating physical function, particularly in older adults. The test has been extensively validated and is commonly used to establish age-specific normative performance benchmarks [17,27]. During the test, participants were instructed to stand up from a standard chair to a fully extended standing position and then return to a seated position as many times as possible within 30 s. The researcher used a stopwatch to time the test, and participants were asked to fold their arms across their chest to eliminate assistance from the upper body. The number of completed cycles was recorded for each participant. The 30CST proved particularly valuable in assessing lower-limb strength, critical for chair transfers, climbing stairs, and getting in and out of bed. The test provided essential insights into the participants’ functional capacity [28] by reflecting the biomechanical demands of the Sit-to-Stand (STS) movement [29,30]. The results were used to evaluate lower-body strength and endurance, which are vital for maintaining mobility, reducing fall risk, and promoting independence, especially in young and older adults [27,29,30].

### 2.4. Statistical Analysis

An a priori power analysis was conducted using G*Power (version 3.1.9.7) to deter-mine the minimum required sample size. Based on a moderate effect size (r = 0.30), an alpha level of 0.05, and a statistical power of 80%, the required sample size was 84 participants. The final sample of 131 participants exceeded this threshold, ensuring adequate statistical power [31]. Statistical analysis was performed using GraphPad Prism version 10.0 (GraphPad Software, Boston, MA, USA).

The Shapiro–Wilk and Kolmogorov–Smirnov tests were used to assess the normality of the data distribution. Quantitative variables were expressed as the mean and standard deviation for normally distributed data and as the median and interquartile range (Q1; 25th percentile and Q3; 75th percentile) for non-normally distributed data. For comparisons of continuous variables, Student’s *t*-test was applied for normally distributed data, while the Mann–Whitney test was used for non-normally distributed data. The Spearman rank correlation coefficient was used to assess the relationships between variables. All *p*-values were two-tailed, and *p*-values < 0.05 were considered statistically significant.

## 3. Results

**Figure 1:**
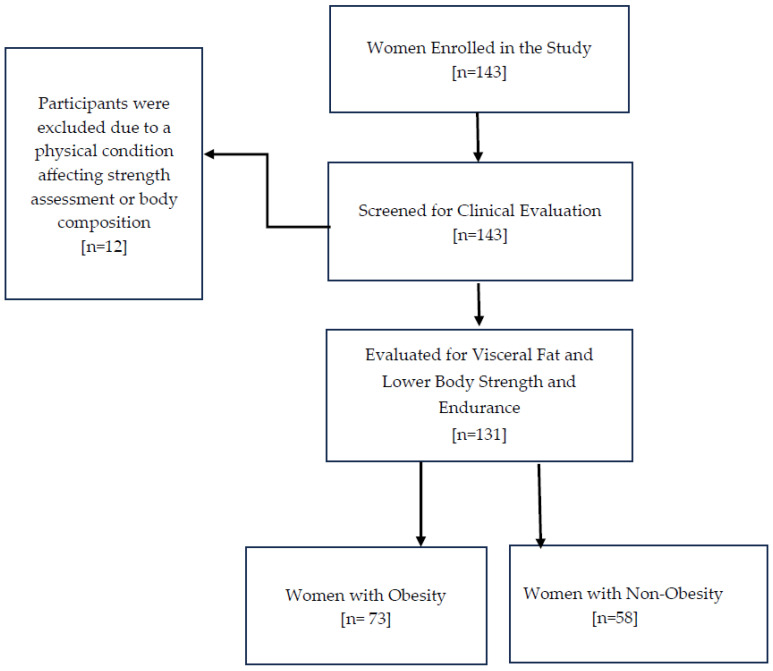
The participant flow chart illustrates the number of women enrolled in the study, outlining those assessed for eligibility, screened for clinical evaluation, and included in the analysis.

**Figure 2:**
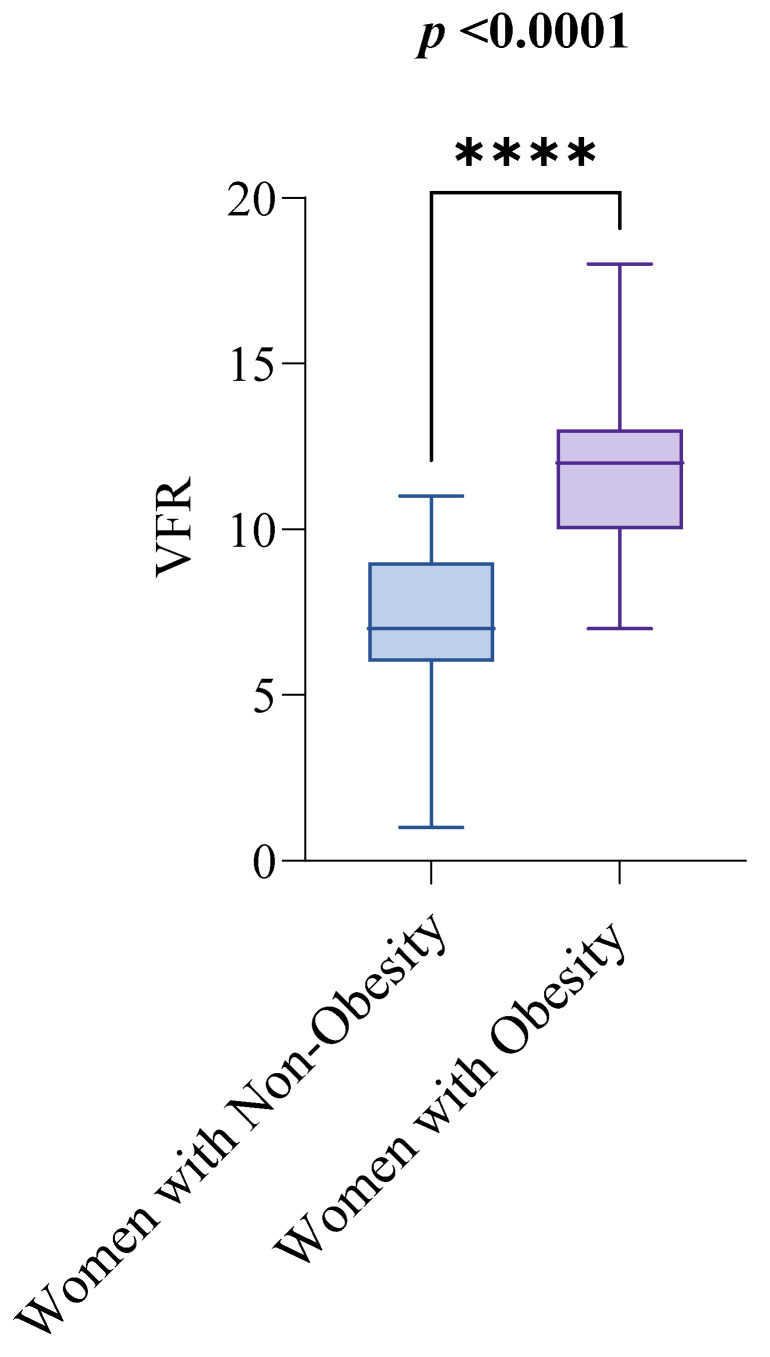
Visceral Fat Rating (VFR) by Obesity Status. Notes: The groups are defined as follows: women with non-obesity (BMI < 30 kg/m^2^) and women with obesity (BMI ≥ 30 kg/m^2^). The Mann–Whitney test was used to compare VFR scores between the two groups. A *p*-value of less than 0.05 indicates statistical significance. Abbreviations: BMI: Body Mass Index; VFR: Visceral Fat Rating. *p* < 0.0001 = ****.

Out of the 143 women initially enrolled in the study, 12 were excluded after screening, leaving 131 women aged 18 to 94 for examination of visceral fat and functional capacity, as shown in Figure 1. Table 1 summarizes the clinical characteristics of the study sample, with the median age being 56 and the interquartile range (IQR) from 45 to 61. Regarding the key variables of interest, the median visceral fat rating (VFR) was 10, with an IQR from 7 to 12, and the median score for the 30 s sit-to-stand test (30CST) was 8, with an IQR from 7 to 10. The study compared these variables between women with obesity (n = 73) and non-obesity (n = 58), using a BMI of 30 kg/m^2^ as the cutoff for obesity diagnosis. The median VFR values were significantly different between the groups: women with non-obesity had a median VFR of 7, with an IQR from 6 to 9, while women with obesity had a median VFR of 12, with an IQR from 10 to 13. This difference was statistically significant (*p* < 0.0001), as shown in Figure 2.

**Figure 3:**
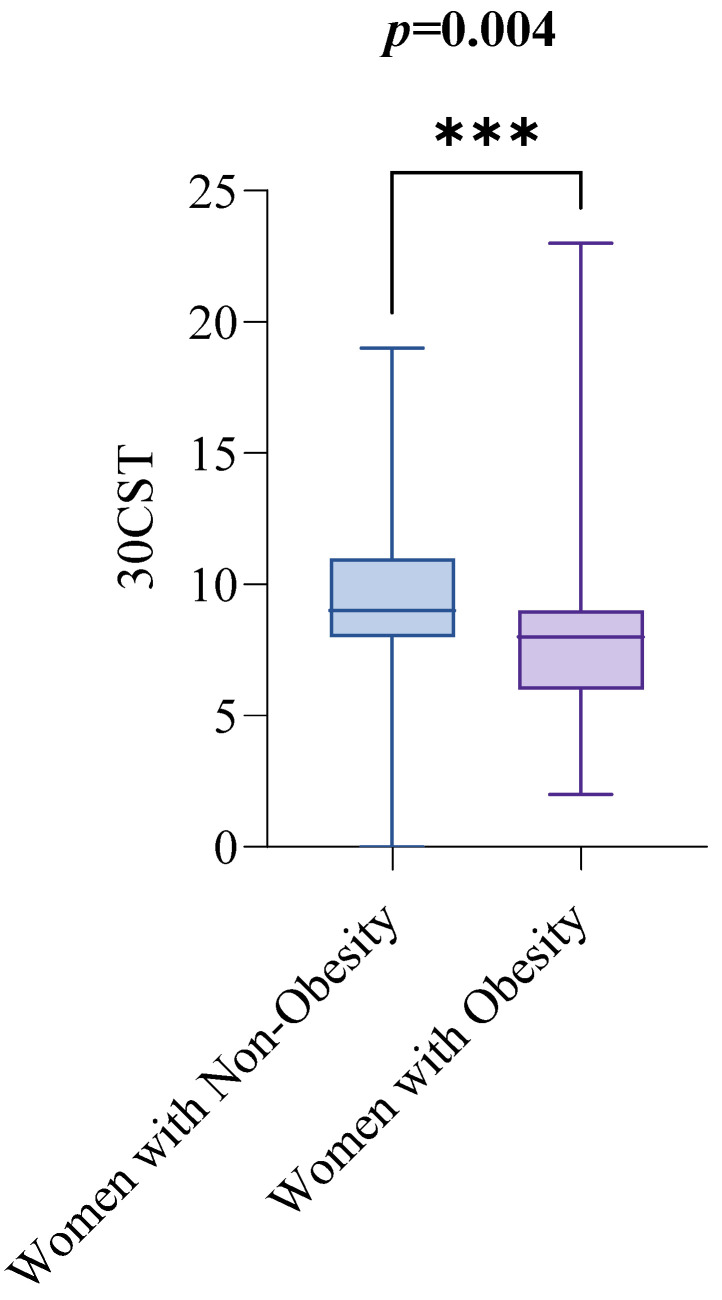
30CST by Obesity Status. Notes: The groups are defined as follows: women with non-obesity (BMI < 30 kg/m^2^) and women with obesity (BMI ≥ 30 kg/m^2^). The Mann–Whitney test was used to compare 300CST scores between the two groups. A *p*-value of less than 0.05 indicates statistical significance. Abbreviations: BMI: Body Mass Index; 30CST: The 30 s sit-to-stand test, also known as the 30 s chair stand test. *p* < 0.001 = ***.

In contrast, women with obesity performed lower on the 30CST than women with non-obesity. The median 30CST score for women with non-obesity was 9, with an IQR from 8 to 11, while for women with obesity, the median score was 8, with an IQR from 6 to 9. The difference was statistically significant (*p* = 0.0004), as illustrated in Figure 3.

Spearman’s rank correlation coefficients were computed to explore the relationships between 30 s sit-to-stand test (30CST) performance and various clinical parameters. Table 2 summarizes the statistical significance of these correlations. A key finding was a significant negative correlation between 30CST performance and visceral fat rating (VFR) (r = −0.4106, *p* < 0.0001). This robust inverse relationship suggests that higher visceral fat is strongly associated with poorer lower body functional capacity, as measured by the 30CST. Additional significant negative correlations were observed between 30CST performance and age (r = −0.3135, *p* = 0.0003), indicating that advancing age is related to a decline in functional capacity. Similarly, weight (r = −0.3020, *p* < 0.0001) and BMI (r = −0.3281, *p* = 0.0001) were negatively correlated with 30CST, reinforcing the impact of higher body weight and BMI on functional performance.

Further negative correlations were found with body fat percentage (%BF) (r = −0.2286, *p* = 0.0086), fat mass (FM) (r = −0.2508, *p* = 0.0039), and fat-free mass (FFM) (r = −0.2213, *p* = 0.0111), suggesting that increased body fat and changes in body composition contribute to reduced functional capacity. Height did not significantly correlate with 30CST performance (r = −0.0060, *p* = 0.9449), indicating that stature does not influence functional performance on the 30CST.In summary, these results highlight the significant impact of visceral fat on functional performance, particularly body strength and endurance. Body composition, weight, and BMI also contribute to 30CST performance, while height was not a significant factor.

## 4. Discussions

Body composition and fat distribution vary significantly across ethnic groups, influencing the thresholds used to define low muscle function [6]. This study is the first to explore the relationship between visceral adipose tissue (VAT) and lower-body strength and endurance in Saudi women, a critical area under-researched in this population. While much of the existing literature relies on body mass index (BMI) as an adiposity measure, BMI does not accurately reflect VAT, a key determinant of metabolic and musculoskeletal health [2,4]. Furthermore, prior research has focused mainly on upper-body strength assessments, particularly handgrip strength, while neglecting the importance of lower-body strength in maintaining functional independence and preventing falls.

The results of this study highlight the significant negative association between elevated VAT and lower-body strength and endurance, both of which are essential for mobility, independence, and overall quality of life [15,16,17].

While the role of VAT in metabolic dysfunction—contributing to conditions such as cardiovascular disease, type 2 diabetes, and insulin resistance—is well-established, its effects on musculoskeletal health [1,2,3,4,5,9,32], particularly lower-body strength, remain less explored. This study specifically investigates the association between VAT and lower-body functional capacity across different BMI categories, comparing women with obesity and non-obesity. This study addresses this gap by highlighting how visceral fat accumulation impairs lower-body functional health. The findings suggest that routine VAT assessment should be incorporated into clinical practice, as early detection and intervention may mitigate the detrimental effects of visceral fat on musculoskeletal function, improving health outcomes and reducing fall risk in women.

The 30 s sit-to-stand test (30CST) was employed to assess lower-body strength and endurance, key determinants of functional mobility and independence. The 30CST is a well-established, reliable clinical tool for evaluating lower-limb strength and endurance. It simulates a typical movement pattern in daily activities, providing a quantifiable measure of functional capacity. Lower-body strength is critical in older adults, as deficits increase fall risk, leading to potential injury and prolonged recovery. The 30CST, when used in conjunction with other assessments such as the Five Times Sit-to-Stand Test (5xSST) and the Fullerton Functional Test [18], is a powerful tool for evaluating fall risk and lower-limb function. These tests are strongly correlated with fall risk and are crucial for clinical evaluations of musculoskeletal health [15,16,17,18,29,30,33,34].

Our findings align with previous studies on Saudi women, confirming the high prevalence of obesity and decreased muscle strength. The median BMI in our cohort was 30.67 kg/m^2^, classifying participants as obese, with a corresponding increase in body fat percentage. These results are consistent with studies showing the high prevalence of obesity among Saudi women across various age groups [6,7,8]. Additionally, prior research on muscle strength in Saudi women has shown significantly lower handgrip strength (HGS) compared to international reference values [6]. Approximately 40% of participants had HGS below 16 kg, a threshold that suggests probable sarcopenia, highlighting the need to consider ethnicity and demographic factors when establishing diagnostic criteria for sarcopenia [6].

Interestingly, our analysis identified a negative correlation between fat-free mass (FFM) and lower-body strength, which contrasts with the usual positive relationship between lean mass and muscle function. This unexpected result may reflect the complex distinction between muscle quantity and quality; intramuscular fat infiltration (myosteatosis) can impair muscle performance despite preserved or even increased lean mass. In individuals with obesity, elevated FFM may partly represent non-contractile tissue, diminishing true muscle function. These findings highlight the need to assess muscle quality alongside quantity in future studies to better elucidate their respective impacts on strength and functional capacity.

Our findings align with Muollo et al. (2025) [35], who showed a strong correlation between handgrip strength (HGS) and the 30 s chair stand test (30CST) in older women. The 30CST demonstrated superior sensitivity and specificity for detecting mobility impairment compared to HGS and the arm curl test, confirming its value for assessing lower-extremity strength and endurance. Aljawini et al. found significantly reduced HGS in women with obesity, prompting investigation into visceral adiposity’s impact on muscle function [6]. Since BMI poorly reflects fat distribution, particularly visceral fat, its clinical utility is limited. The relationship between VAT, measured by BIA, and lower-limb function in women remains underexplored. Our data suggest elevated VAT impairs lower-extremity strength, increasing risk of mobility decline and falls. These findings support routine use of the 30CST in clinical practice, especially for women with obesity, to prevent functional decline.

BIA, employed in this study to assess visceral adipose tissue (VAT), is a practical, non-invasive, and low-cost alternative to imaging [12,24,36]. With rising rates of sarcopenic obesity and VAT-related cardiometabolic risk, accessible assessment tools are essential [10]. While MRI remains the gold standard, its high operational and logistical demands limit routine use [1,2]. BIA offers a feasible option for VAT estimation and risk stratification in clinical practice.

A recent study by Hoffman et al. validated BIA as a practical alternative to MRI for quantifying VAT [36]. By combining BIA with ultrasound-derived subcutaneous fat measurements, they developed a sex-specific equation to estimate VAT. The study found that BIA provides reliable estimates of VAT, with performance comparable to MRI [36], highlighting its potential as a non-invasive, cost-effective tool for both clinical and research settings. This reinforces the value of BIA in improving the clinical management of metabolic and cardiovascular risks associated with visceral fat.

Myosteatosis, fat infiltration into muscle, is linked to muscle dysfunction, obesity, insulin resistance, and chronic inflammation [37,38]. It reduces muscle strength and worsens sarcopenia even without major muscle loss [39]. Visceral fat drives lipolysis and free fatty acid release, promoting intramuscular fat and impairing muscle and metabolic health.

Our cohort’s median age was 56, representing perimenopausal and postmenopausal women. Menopause is associated with substantial changes in fat distribution, including increased visceral and subcutaneous fat [40]. Abildgaard et al. reported that postmenopausal women exhibit larger adipocytes, increased inflammation, fibrosis in subcutaneous fat, more significant visceral fat accumulation, and reduced insulin sensitivity [40]. These metabolic changes elevate this population’s risk of metabolic and cardiovascular diseases.

Adipose tissue infiltration into muscle mass leads to alterations in muscle composition [37], referred to as “muscle quality” [10,22,41]. These changes in muscle quality are critical determinants of muscle function and clinical outcomes [42,43]. Muscle quality and the extent of adipose infiltration should be integral to assessing functional capacity, mobility, and prognosis in individuals with obesity. Our study underscores the need for a comprehensive approach to managing adiposity-related comorbidities, focusing on the interplay between visceral adiposity, myosteatosis, and muscle dysfunction.

Taken together, this study highlights significant associations between visceral adipose tissue (VAT) and lower-body strength and endurance in Saudi women, emphasizing the utility of bioelectrical impedance analysis (BIA) as a practical, non-invasive, and cost-effective tool for VAT assessment. Unlike BMI, which does not capture fat distribution, BIA provides a more precise estimate of VAT, a key factor in metabolic and musculoskeletal health. The 30 s sit-to-stand test (30CST), assessing lower-body function, further underscores its relevance for evaluating functional independence, mobility, and fall risk. A major strength of this study is the use of clinically feasible tools, facilitating integration into routine practice. However, this exploratory study included a modest sample of Saudi women from a single region, limiting the generalizability of the findings. Its cross-sectional design prevents causal inference regarding the relationship between visceral adiposity and lower-body function. Visceral adipose tissue was estimated using the BIA-derived Visceral Fat Rating (VFR), a practical but less precise alternative to gold-standard imaging methods such as MRI or DXA. Important factors including physical activity, diet, and menopausal status were not assessed. Despite these limitations, the study provides valuable preliminary data that can inform future larger, longitudinal research utilizing advanced imaging techniques and more diverse populations.

Combining BIA-derived VAT measurements with functional assessments like the 30CST holds promise for early identification of metabolic and musculoskeletal impairments, enabling timely intervention in at-risk populations.

## 5. Conclusions

Higher visceral adipose tissue (VAT) was associated with reduced lower-body strength and endurance in women. These findings highlight the potential clinical utility of VAT assessment, as elevated levels may indicate risk for functional decline. Although not a gold-standard, bioelectrical impedance analysis (BIA) offers a practical and accessible approach when advanced imaging modalities such as MRI or DXA are not feasible. Integrating VAT evaluation into routine clinical practice may enable early identification of at-risk individuals and guide interventions to maintain musculoskeletal function, mobility, and quality of life, particularly in women affected by obesity or aging.

## Figures and Tables

**Table 1:** Descriptive Statistics of Study Participants.

Parameters for All Females [n = 131]	
**Age (years)**	56.00 (45.00, 61.00)
**Anthropometrics**	
Weight (kg)	74.00 (64.70, 89.80)
Height (cm)	156.30 (6.29)
BMI (kg/m^2^)	30.67 (27.25, 36.51)
**Body Composition**	
%BF	40.20 (35.50, 43.30)
FM (kg)	30.50 (23.30, 37.70)
FFM (kg)	44.70 (41.10, 51.00)
**Research Variables**	
VFR	10.00 (7.00, 12.00)
30CST	8.00 (7.00, 10.00)

Notes: Continuous variables are expressed as mean and standard deviation (SD) for normally distributed data or median and interquartile range (25,75%) for non-normally distributed data. Abbreviations: BMI: body mass index; %BF: percentage body fat; FM: fat mass; FFM: fat-free mass; VFR: visceral fat rating; 30CST: The 30 s sit-to-stand test, also known as the 30 s chair stand test.

**Table 2:** Bivariate correlation of 30CST with study parameters in the total sample.

Parameter	Correlation Coefficient (r)	95% Confidence Interval	*p* Value	*p* Value Summary
Age	−0.3135	(−0.4643, 0.1450)	0.0003	***
Weight (kg)	−0.3020	(−0.4121, −0.1833)	<0.0001	****
Height (cm)	−0.0060	(−0.1824, 0.1706)	0.9449	ns
BMI (kg/m^2^)	−0.3281	(−0.4769, −0.1609)	0.0001	***
%BF	−0.2286	(−0.3894, 0.0543)	0.0086	**
FM (kg)	−0.2508	(−0.4092, −0.0777)	0.0039	**
FFM (kg)	−0.2213	(−0.3785, −0.0517)	0.0111	*
VFR	−0.4106	(−0.5474, −0.2524)	<0.0001	****

Notes: Spearman’s rank correlation coefficient (r) evaluated the strength and direction of the association between the 30CST and each parameter in the total sample. The correlation is significant at the 0.05 level * (two-tailed). Significant *p*-values are indicated as follows: *p* < 0.0001 = ****, *p* < 0.001 = ***, *p* < 0.01 = **, *p* < 0.05 = *, and *p* ≥ 0.05 = ns (not significant). Abbreviations: BMI: body mass index; %BF: percentage body fat; FM: fat mass; FFM: fat-free mass; VFR: visceral fat rating; 30CST: The 30 s sit-to-stand test, also known as the 30 s chair stand test.

## Data Availability

The data are not publicly available due to ethical and institutional restrictions to protect the participants’ confidentiality, as per the regulations of the institutions’ IRB.
